# Cardiovascular disease risk among transgender women living with HIV in the United States

**DOI:** 10.1371/journal.pone.0236177

**Published:** 2020-07-20

**Authors:** Bennett J. Gosiker, Catherine R. Lesko, Ashleigh J. Rich, Heidi M. Crane, Mari M. Kitahata, Sari L. Reisner, Kenneth H. Mayer, Rob J. Fredericksen, Geetanjali Chander, William C. Mathews, Tonia C. Poteat

**Affiliations:** 1 Department of Epidemiology, Johns Hopkins Bloomberg School of Public Health, Baltimore, MD, United States of America; 2 School of Population and Public Health, Faculty of Medicince, University of British Columbia, Vancouver, BC, Canada; 3 Department of Medicine, Division of Allergy and Infectious Diseases, University of Washington, Seattle, WA, United States of America; 4 The Fenway Institute, Boston, MA, United States of America; 5 Division of General Pediatrics, Boston Children’s Hospital, Boston, MA, United States of America; 6 Department of Pediatrics, Harvard Medical School, Boston, MA, United States of America; 7 Department of Epidemiology, Harvard TH Chan School of Public Health, Boston, MA, United States of America; 8 Department of Medicine, Beth Israel Deaconess Medical Center, Harvard Medical School, Boston, MA, United States of America; 9 Department of Medicine, Johns Hopkins School of Medicine, Baltimore, MD, United States of America; 10 School of Medicine, University of California San Diego, San Diego, CA, United States of America; 11 Department of Social Medicine, University of North Carolina School of Medicine, Chapel Hill, NC, United States of America; British Columbia Centre for Excellence in HIV/AIDS, CANADA

## Abstract

**Background:**

Transgender women (TW) are disproportionately affected by both HIV and cardiovascular disease (CVD).

**Objectives:**

We aim to quantify prevalence of elevated predicted CVD risk for TW compared to cisgender women (CW) and cisgender men (CM) in HIV care and describe the impact of multiple operationalizations of CVD risk score calculations for TW.

**Design:**

We conducted a cross-sectional analysis of patients engaged in HIV care between October 2014 and February 2018.

**Setting:**

The Centers for AIDS Research Network of Integrated Clinical Systems, a collaboration of 8 HIV clinical sites in the United States contributed data for this analysis.

**Patients:**

221 TW, 2983 CW, and 13467 CM.

**Measurements:**

The measure of interest is prevalence of elevated 10-year cardiovascular disease risk based on ACC/AHA Pooled Cohort Risk Assessment equations (PCE) and the Framingham Risk Score (FRS), calculated for TW by: birth-assigned sex (male); history of exogenous sex hormone use (female/male); and current gender (female).

**Results:**

Using birth-assigned sex, the adjusted prevalence ratio (aPR) was 2.52 (95% CI: 1.08,5.86) and 2.58 (95% CI: 1.71,3.89) comparing TW to CW, by PCE and FRS, respectively. It was 1.25 (95% CI: 0.54,2.87) and 1.25 (95% CI: 0.84,1.86) comparing TW to CM, by PCE and FRS, respectively. If TW were classified according to current gender versus birth-assigned sex, their predicted CVD risk scores were lower.

**Limitations:**

PCE and FRS have not been validated in TW with HIV. Few adjudicated CVD events in the data set precluded analyses based on clinical outcomes.

**Conclusions:**

After adjustment for demographics and history of HIV care, prevalence of elevated CVD risk in TW was similar to CM and equal to or higher than in CW, depending operationalization of the sex variable. Future studies with CVD outcomes are needed to help clinicians accurately estimate CVD risk among TW with HIV.

## Introduction

Transgender women (TW) are disproportionately affected by HIV infection and related comorbidities [1–4]. Globally, 19% of TW are living with HIV and TW have 49 times greater odds of HIV-infection compared to cisgender adults of reproductive age in their respective countries [5]. In the United States, the most recent HIV prevalence estimate among TW is 14% [1] compared with 0.3% in the general population [6].

People living with HIV have a higher prevalence of cardiovascular disease (CVD) than persons without HIV, related to both higher prevalence of traditional risk behaviors for CVD such as smoking [7, 8], and the effects of HIV itself [9, 10]. HIV infection is associated with chronic inflammation and elevated burden of CVD risk factors such as diabetes [11]. Specific antiretroviral therapy (ART) regimens have also been associated with higher risk of CVD [12, 13].

In the general population, TW experience disparities in prevalence of CVD risk factors, rates of events (e.g., myocardial infarction, stroke, venous thromboembolism) [7, 8, 11], and potentially risk of CVD-related mortality [14, 15] compared to their cisgender counterparts. This elevated CVD burden is a function of multilevel factors, including biological (e.g., exogenous sex-hormone use), interpersonal (e.g., gender-based stigma) and socio-structural (e.g., discrimination) [3, 14–16].

TW living with HIV are uniquely vulnerable to CVD morbidity as a result of potentially mutually-reinforcing HIV-related and gender-identity-related risks. For example, gender-related stigma contributes to sub-optimal: retention in care [15], ART adherence, and viral suppression for TW living with HIV [17], all factors that may impact CVD risk. Gender affirming hormone therapy (GAHT), including exogenous sex-hormone use, is an important part of healthcare for many TW [18–20]. GAHT can increase ART adherence and viral suppression for TW living with HIV [18–20] which may improve outcomes [21]. However, exogenous estrogen use affects inflammation and immune function, potentially conferring increased CVD risk among TW using GAHT [2, 9, 12, 14, 16, 19, 22]. In short, both HIV and GAHT play a role in CVD [23, 24].

Despite the unique vulnerability to CVD-related morbidity and mortality faced by TW living with HIV, data on CVD risk among TW living with HIV are lacking [14]. The objectives of this study were to quantify elevated CVD risk prevalence for TW compared to cisgender women (CW) and cisgender men (CM) engaged in HIV care and describe the impact of multiple operationalizations of CVD risk scores for TW.

## Methods

### Study sample

This study used data from the Centers for AIDS Research Network of Integrated Clinical Systems (CNICS), a multi-site collaboration of clinical sites that deliver care to people living with HIV across the U.S. [25]. Briefly, CNICS includes adults engaged in HIV care at one of 8 medical centers who consent to share their data (>90% of eligible patients). Full details of the cohort have been published elsewhere [25]. Comprehensive clinical data were collected through electronic medical records and other data systems including patient demographic characteristics, diagnoses, prescribed medications, and laboratory results. CNICS data undergo quality assessment, are harmonized in a central repository, and are updated on a quarterly basis [25]. Medications include those prescribed by HIV providers and other clinicians and medications that are reported to the HIV provider during an HIV care visit and entered into the medication list. Patient-reported measures and outcomes are collected via Computer-Assisted Self-Interview (CASI) on touch screen tablets at routine clinic visits approximately every 4–6 months [26]. For this analysis, we included patients who were alive and engaged in any medical care at a CNICS site as of the site-specific administrative censoring date (ranged from October 2014-February 2018). Engagement in care was defined broadly as having a CD4 cell count or viral load recorded in the most recent two years of data collection at each site. This definition of engagement in care is intended to be more sensitive and less specific than definitions of engagement in care used for describing the HIV care continuum. Here, engagement in care is intended as a marker of a patient’s potential to have any recent CVD risk factors recorded in the medical record, and to not be completely lost to the CNICS clinic.

### Study measures

The primary independent variable of interest was gender identity, categorized as: CW, CM, TW, and transgender men. Transgender status was captured using different approaches across the contributing cohorts. TW included participants who were assigned male sex at birth and had a diagnosis of gender dysphoria, were taking feminizing hormones, and/or were identified as women or TW by self-report or provider report in the medical record. We excluded 14 transgender men and 1 intersex individual from the analysis as there were too few persons to conduct meaningful subanalyses for these groups.

Our outcome, CVD risk score, is traditionally calculated based on birth-assigned sex, classified as either female or male. Guidelines do not exist for calculating CVD risk scores for transgender persons. As such, we assessed the impact of operationalizing the “sex” variable in calculating CVD risk scores for TW in three ways: 1) by sex assigned at birth (male for all TW); 2) by exogenous sex hormone use—using female for TW who had used exogenous sex hormones and male for TW who had not used exogenous sex hormones, under the hypothesis that hormonal profile was the ‘sex’ construct being used to predict CVD risk; and 3) by current gender (female for all TW). Here, we use ‘female’ to indicate sex assigned for risk score calculation, based on current gender identity for TW (‘woman’). Exogenous sex hormone use was determined by the presence of prescriptions for any progestin or estrogen-containing regimen during CNICS enrollment.

We calculated 10-year predicted CVD risk based on American College of Cardiology/American Heart Association Pooled Cohort Risk Assessment Equations published in 2014 (PCE risk score) and the Framingham Risk Score (FRS) [27, 28]. The PCE is calculated based on age, sex (operationalized as described above), race, systolic blood pressure, total cholesterol, high-density lipoprotein (HDL) cholesterol, anti-hypertensive medication use, diabetes status, and smoking status [27]. The FRS includes all of the variables in the PCE with the exception of race. The PCE risk score is a measure of the 10-year risk of the occurrence of nonfatal myocardial infarction, coronary heart disease death, or fatal or nonfatal stroke [27]. The FRS is a more encompassing measure of the 10-year risk of occurrence of coronary heart disease, including coronary death, myocardial infarction, coronary insufficiency, angina, cerebrovascular events (including hemorrhagic stroke, ischemic stroke, and transient ischemic attack), peripheral artery disease (intermittent claudication), and heart failure [28].

Race was classified as Black or non-Black for calculation of risk scores. Systolic blood pressure, total cholesterol and HDL cholesterol (whether fasting or random) were those most recently recorded prior to the site-specific administrative censoring date. Anti-hypertensive use was based on prescriptions. Diabetes was defined as ever having had a recorded hemoglobin A1c (HbA1c) measurement ≥6.5%, ever having been prescribed a medication specific for diabetes (S1 Table), ever having received a diagnosis of diabetes and been prescribed a diabetes management medication (S1 Table), or having had ≥2 laboratory measures of blood glucose (random or fasting) ≥200 mg/dL. Current tobacco smoking was determined from the clinical assessment of patient-reported measures and outcomes. Elevated CVD risk was classified as having a predicted 10-year PCE risk score or FRS greater than or equal to 7.5%.

We report both crude and adjusted estimates of the prevalence of elevated PCE risk score and FRS for TW compared to CW and CM. We adjusted estimates for race/ethnicity, age, viral suppression at most recent visit (where <400 copies/mL was classified as virally suppressed), most recent CD4 cell count, study site, cumulative years of exposure to any antiretroviral therapy (ART), and exposure to any abacavir-containing ART regimen. Abacavir-containing regimens were included due to their association with increased risk for myocardial infarctions [13]. For adjustment, race/ethnicity was classified as Hispanic, non-Hispanic Black (Black), non-Hispanic white (white), or non-Hispanic, non-Black, non-white race (other). For the purposes of calculating CVD risk, we multiply imputed a race for everyone (see below).

Adjustment was undertaken to determine how much of any observed disparities in PCE risk score and FRS comparing TW to CW or CM would remain if the population of TW versus CW or CM in care for HIV were balanced on the distribution of the adjustment variables. Some of the adjustment variables are predictors in the PCE risk score (race, age) and FRS (age). Adjusted estimates are thus interpreted as the disparity in predicted CVD risk associated with the other predictors in each model. Adjusting for clinical indicators such as viral suppression and CD4 count should only change prevalence ratios in so far as they are associated with the other components of the CVD risk predictors.

### Statistical analysis

We estimated prevalence ratios (PR) for elevated PCE risk score and FRS (≥7.5%) comparing TW, CW and CM using log-binomial models. Crude prevalence ratios describe the excess of the predicted CVD risk burden borne by TW. Adjustment was accomplished using inverse probability weighting [29, 30]. We estimated the denominator of the weights using logistic regression models to generate the predicted probability of being a TW in our sample conditional on race/ethnicity, age, viral suppression at most recent visit, most recent CD4 cell count, study site, cumulative years of exposure to ART, and an indicator of any exposure to abacavir-containing ART. We stabilized the weights by the marginal probability of being ones’ gender identity in the sample. We used robust standard error estimates for the PRs [31] to account for uncertainty in weight estimation.

We used multiple imputation with chained equations to handle missing values for the components of the PCE risk score, FRS, and adjustment variables [32, 33]. We imputed missing values 20 times, conducted all analyses within each of the 20 imputed datasets, and combined estimates using Rubin’s rules [34]. The data analysis presented was performed in SAS, version 9.4.

## Results

The study sample included 221 TW, 2,983 CW, and 13,467 CM. Sample median age was 39 years (interquartile range [IQR]: 31,47), and TW in the sample were slightly younger with a median age of 36 years (IQR: 28,42). Median CD4+ cell count was 603 cells/μL (IQR: 398,835) for the entire sample, with TW having the lowest median CD4+ cell count of 560 cells/μL (IQR: 358,832). The majority of patients (89%) were virologically suppressed at their most recent visit. TW had slightly less ART exposure with a median of 5 years (IQR: 2,10) as compared to CM and CW who had a median of 7 years (IQR: 3,12) (Table 1). Forty-three percent of TW (n = 94) had a history of exogenous sex hormone use (n = 82 estrogen; n = 2 progestin; and n = 31 combination estrogen/progestin).

**Table 1:** Baseline demographic and HIV-related characteristics of 16,671 adults alive and in care at a CNICS site as of the site-specific administrative censoring date, October 2014-February 2018*.

	Cisgender Men (N = 13467)	Cisgender Women (N = 2983)	Transgender Women (N = 221)	Total (N = 16671)
**Age**^**†**^	39 (31,47)	39 (32, 47)	36 (28, 42)	39 (31,47)
**Race/Ethnicity**^**‡**^^,^^**§**^				
**Black**	4492 (34%)	1967 (66%)	97 (45%)	6556 (40%)
**White**	6137 (46%)	632 (21%)	40 (18%)	6809 (41%)
**Hispanic**	2003 (15%)	268 (9%)	64 (29%)	2335 (14%)
**Other**	677 (5%)	99 (3%)	17 (8%)	793 (5%)
**CD4 (cells/μL)**^**||**^	593 (393,814)	658 (426,920)	560 (358,832)	603 (398,835)
**ART Exposure (Years)**	7 (3,12)	7 (4,12)	5 (2,10)	7 (3,12)
**Ever on an abacavir-containing ART regimen**^**‡**^	4147 (31%)	1121 (38%)	64 (29%)	5332 (32%)
**Viral Suppression (HIV RNA < 400 copies/mL)**^**‡**^^,^^**¶**^	11967 (89%)	2621 (88%)	181 (82%)	14769 (89%)

*Abbreviations: HIV, human immunodeficiency virus; ART, antiretroviral therapy

^†^Median (IQR) unless otherwise specified

^‡^N (%)

^§^Missingness of Race/Ethnicity: Cisgender Men: 158 (1%), Cisgender Women: 17 (<1%), Transgender Women: 3 (1%), Total: 178 (1%)

^||^Missingness of CD4: Cisgender Men: 11 (<1%), Cisgender Women: 1 (<1%), Transgender Women: 0 (0%), Total: 12 (<1%)

^¶^Missingness of Viral Suprression: Cisgender Men: 40 (<1%), Cisgender Women: 3 (<1%), Transgender Women: 3 (1%), Total: 12 (<1%)

The median 10-year PCE risk scores were 2.3% (IQR: 0.8% - 5.8%) and 1.1% (IQR: 0% -4.0%) for CM and CW respectively. For TW, the median 10-year PCE risk score was 2.4% (IQR: 0.8% - 5.0%) when calculated using birth sex (male), 1.9% (IQR: 0.6% - 3.7%) when calculated based on exogenous sex hormone use, and 0.9% (IQR: 0.3% - 3.0%) when calculated using current gender (female) (Table 2). The median FRSs were 5.6% (IQR: 2.8% - 11.2%) and 3.9% (IQR: 2.0% - 7.3%) for CM and CW respectively. For TW, the median 10-year FRS was 4.7% (IQR: 2.8% - 7.9%) when calculated using birth sex (male), 3.9% (IQR: 2.0% - 6.7%) when calculated based on exogenous sex hormone use, and 2.8% (IQR: 1.7% - 4.5%) when calculated using current gender (female). TW had higher prevalence of being treated for hypertension than CM and CW and lower prevalence of a history of diabetes (Table 2).

**Table 2:** CVD risk score predictors and median and IQR of CVD risk score among 16,671 Adults Alive and in Care at a CNICS Site as of the Site-Specific Administrative Censoring Date, October 2014-February 2018*.

	Cisgender Men (N = 13467)	Cisgender Women (N = 2983)	Transgender Women (N = 221)	Total (N = 16671)
**Race** ^**†**^^,^^**‡**^^,^^**§**^				
**Black**	4522 (35%)	1976 (67%)	101 (49%)	6599 (41%)
**Non-Black**	8550 (65%)	970 (33%)	107 (51%)	9627 (59%)
**Systolic Blood Pressure (mmHg)**^**||**^^,^ ^**¶**^	126 (117,136)	126 (114,138)	122 (113,131)	126 (117,136)
**Diabetes**	1651 (12%)	596 (20%)	18 (8%)	2265 (14%)
**Treatment for Hypertension**	5338 (40%)	1629 (55%)	162 (73%)	7129 (43%)
**HDL Cholesterol (mg/dL)**^**||**^^,^^**^	44 (36,53)	52 (42,64)	49 (38,56)	45 (37,55)
**Total Cholesterol (mg/dL)**^**||**^^,^^**††**^	170 (145,197)	177(153,205)	168 (147,192)	171 (147,198)
**Current Tobacco Smoker**^**‡‡**^	3129 (34%)	691 (35%)	55 (37%)	3875 (34%)
**Median (IQR) 10-year PCE risk score based on:**				
**Birth sex**	2.3 (0.8, 5.8)	1.1 (0, 4.0)	2.4 (0.8, 5.0)	2.1 (0.7, 5.5)
**History of sex hormone use**	-	-	1.9 (0.6, 3.7)	2.1 (0.7, 5.5)
**Current gender**	-	-	0.9 (0.3, 3.0)	2.1 (0.7, 5.5)
**Median (IQR) 10-year FRS based on:**				
**Birth sex**	5.6 (2.8, 11.2)	3.9 (2.0, 7.3)	4.7 (2.8, 7.9)	4.7 (2.8, 9.4)
**History of sex hormone use**	-	-	3.9 (2.0, 6.7)	4.7 (2.8, 9.4)
**Current gender**	-	-	2.8 (1.7, 4.5)	4.7 (2.8, 9.4)

*Abbreviations: CVD, cardiovascular disease; FRS, Framingham Risk Score; PCE, Pooled Cohort Equation; HDL, High Density Lipoprotein; SE, Standard Error

^†^N(%) unless otherwise specified

^‡^Number missing is greater than in Table 1, reflecting persons who did not report a race but did report Hispanic ethnicity; see text for more details

^§^Missingness of Race: Cisgender Men: 395 (3%), Cisgender Women: 37 (1%), Transgender Women: 13 (6%), Total: 445 (3%)

^||^Median (IQR)

^¶^Missingness of Systolic Blood Pressure: Cisgender Men: 15 (<1% %), Cisgender Women: 13 (<1% %), Transgender Women: 1 (<1% %), Total: 29 (<1%)

**Missingness of HDL Cholesterol: Cisgender Men: 575 (4%), Cisgender Women: 127 (4%), Transgender Women: 6 (3%), Total: 708 (4%)

^††^Missingness of Total Cholesterol: Cisgender Men: 399 (3%), Cisgender Women: 103 (3%), Transgender Women: 4 (2%), Total: 506 (3%)

^‡‡^Missingness of Current Tobacco Smoking Status: Cisgender Men: 4175 (31%), Cisgender Women: 1004 (34%), Transgender Women: 73 (33%), Total 5252 (32%).

In adjusted analyses, point estimates for the prevalence of elevated FRS and PCE risk scores was always higher for TW compared to CW regardless of how risk scores were calculated for TW. The magnitude of the increased predicted risk of CVD was similar for PCE risk scores and FRS. Adjusted prevalence ratios (aPR) for elevated PCE risk score for TW compared to CW were 2.52 (95% confidence interval [CI]: 1.08–5.86) when TW were classified according to birth sex (male) and 1.52 (95% CI: 0.38, 6.08) when TW were classified according to current gender (female). For elevated FRS, the aPR was 2.58 (95% CI: 1.71, 3.89) when TW were classified according to birth sex, and 1.26 (95% CI: 0.53, 2.96) when TW were classified according to current gender (Table 3).

**Table 3:** Associations Between Sex and Gender and Elevated CVD Risk Scores Among 221 Transgender Women, 2,983 Cisgender Women, and 13,467 Cisgender Men Alive and Engaged in Continuity HIV Care in the CNICS, 2014–2018 (or contributing cohort-specific administrative censoring date)*.

	Pooled Cohort Equation (PCE)				Framingham Risk Score (FRS)			
	Crude		Adjusted*		Crude		Adjusted*	
	Prevalence Ratio	95% CI	Prevalence Ratio	95% CI	Prevalence Ratio	95% CI	Prevalence Ratio	95% CI
**Transgender Women Classified by Birth Sex**^†^ **Compared to:**								
Cisgender Women	1.03	(0.65, 1.63)	2.52	(1.08, 5.86)	1.06	(0.79, 1.43)	2.58	(1.71, 3.89)
Cisgender Men	0.77	(0.49, 1.21)	1.25	(0.54, 2.87)	0.70	(0.53, 0.94)	1.25	(0.84, 1.86)
**Transgender Women Classified According to History of Exogenous Sex Hormone Use**^†^ **Compared to: PresentFemalesb** **by Present Sex (Female)**								
Cisgender Women	0.75	(0.44, 1.28)	2.14	(0.79, 5.81)	0.77	(0.54, 1.10)	1.77	(0.97, 3.24)
Cisgender Men	0.56	(0.33, 0.96)	1.06	(0.40, 2.85)	0.51	(0.36, 0.72)	0.86	(0.47, 1.55)
Transgender Women Classified by Current Gender^*^ **Compared to:**								
Cisgender Women	0.35	(0.14, 0.84)	1.52	(0.38, 6.08)	0.48	(0.30, 0.76)	1.26	(0.53, 2.96)
Cisgender Men	0.26	(0.11, 0.62)	0.75	(0.19, 2.99)	0.32	(0.20, 0.50)	0.61	(0.26, 1.42)

*Adjusted for race, age, most recent viral load (<400 versus ≥400 copies/mL), most recent CD4 cell count, study site, cumulative years of exposure to antiretroviral therapy, and any history of exposure to an abacavir-containing antiretroviral therapy regimen

^†^In calculations of PCE risk score and FRS

Prevalence of elevated PCE risk scores and FRS was higher for TW compared to CM when TW were classified according to birth sex (male). The aPR of elevated PCE risk score was 1.25 (95% CI: 0.54, 2.87) and the aPR of elevated FRS was 1.25 (95% CI: 0.84, 1.86). Prevalence of elevated predicted CVD risk was lower for TW compared to CM if TW were classified according to current gender (female). The aPR of elevated PCE risk score was 0.75 (95% CI: 0.19, 2.99) and the aPR of elevated FRS was 0.61 (95% CI: 0.26, 1.42). When TW were classified according to use of exogenous sex hormones it resulted in aPRs close to the null: an aPR of 1.06 (95% CI: 0.40, 2.85) for elevated PCE risk score and an aPR of 0.86 (95% CI: 0.47, 1.55) for elevated FRS (Table 3).

## Discussion

This study used the FRS and PCE to estimate the prevalence of elevated predicted CVD risk for TW compared to CW and CM in HIV care and described the impact of multiple operationalizations of sex in CVD risk score calculations for TW. After adjustment for demographics and history of HIV care, prevalence of elevated CVD risk in TW was similar to CM and equal to or higher than in CW, depending operationalization of the sex variable. Adjusted estimates were meaningfully different from crude estimates for some estimated prevalence ratios, particularly when using current gender for the PCE. That is, although in adjusted analyses TW had elevated predicted risk of CVD compared to CW, their crude predicted risk of CVD was lower in almost all instances, likely as a function of their younger age and lower prevalence of diabetes (Table 2).

Estimated 10-year CVD risk was highest for TW when classifying them according to birth-assigned sex (male) and lowest when classifying them according to current gender (female). This is not surprising because both the PCE and FRS attribute greater risk to persons classified as male [27, 28]. The sample average risk scores for TW was between these two extremes when calculated based on exogenous sex hormone use. After adjustment, TW had a prevalence of elevated CVD risk comparable to CM. TW consistently had an equivalent or higher prevalence of elevated CVD risk compared to in CW in adjusted analyses.

One of the only other existing studies exploring CVD among TW (n = 23) and CM (n = 92) living with HIV found elevated levels of anemia, depression, HCV infection, and poor HIV control [35]. Many of the traditional CVD risk factors measured in the study were not different among TW and CM. Our larger sample of TW (n = 221) had a lower prevalence of viral suppression and diabetes, lower CD4 counts, and higher prevalence of treated hypertension than CW and CM (Table 2, Table 3).

In one study of TW (N = 214) and age-matched CM and CW without HIV in Belgium, TW had 4.2% higher prevalence of prior MI than CM, and 18.7% higher prevalence of prior MI than CW. TW also had higher prevalence of history of stroke or cerebrovascular disease, obesity, and diabetes (4.2% versus 0.6% in CM and 1.5% in CW) [11]. The largest difference in prevalence of a CVD risk factor in our sample was treatment for hypertension; 73% of TW had a history of treatment for hypertension compared with 40% of CM and 55% of CW. The differences in the prevalence of CVD risk factors in our study sample and the sample from Belgium may reflect differences in cultural norms related to CVD risk factors and gender, the influence of HIV, or socioeconomic factors that intersect with the HIV epidemic.

We found no other publishd studies that assessed cardiovascular disease risk or events among TW living with HIV and accounted for GAHT use. Studies of the effect of GAHT use on CVD risk among TW without HIV have varying results, with some evidence of increases in thromboembolic events [36, 37]. Among patients who received care in the Kaiser Permanente health care system in Georgia or California, the adjusted hazard ratio of venous thromboembolism in TW using exogenous sex hormones was 3.2 (95% CI: 1.5,6.5) compared to CM and 2.5 (95% CI: 1.2–5.0) compared to CW. Incidence of ischemic stroke and MI were similar across comparison grops [38]. Neither the PCE nor FRS were designed to capture risk for thromboembolic events [27, 28]. Other studies have shown elevated endothelial activation, inflammatory biomarkers, and brachial artery diameter as indicators of elevated CVD risk among TW [39–41].

In this study, predicted CVD risk for TW was not appreciably higher than for CM. However, CVD risk as quantified by the PCE risk score and FRS do not capture the potential effects of CVD risk factors unique to TW including but not limited to exogenous sex hormone use and stress related to violence or stigma. Exogenous estrogen use affects inflammation and immune function [2, 9, 12, 14, 16, 19, 22] as does stress [42–45]. Our results should be interpreted cautiously given the absence of CVD endpoints and the notable affect of altering how we operationalized the “sex” variable for TW in the CVD risk equations.

The variation in CVD risk estimates based on the operationalization of “sex” highlights the practical limitations of both the FRS and PCE when used by clinicians who seek to identify and address elevated CVD risk among their TW patients with HIV. It is unclear if the differences in CVD risk found between CW and CM can be attributed to hormonal differences alone or if other sex and/or gender-based factors account for this differences. Therefore, important research questions remain on which “sex” is appropriate to input when attempting to estimate CVD risk among TW via these commonly used calculators.

Importantly, neither the FRS nor PCE have been validated among TW, and the correspondence between predicted risk and actual risk is unknown for this population. The PCE and FRS are useful tools for motivating discussions of CVD prevention for people living with HIV, but were originally developed and calibrated for use in the general population. Persistent immune activation in people living with HIV and increased systemic inflammation is thought to contribute to elevated risk for a variety of cardiovascular disease outcomes [23]. Feinstein et al. assessed the PCE risk score calculation in CNICS and found the best fit for white men, but under-prediction for Black men, Black women, and white women [24]. The proportion of Black participants in this study varied by gender, making up 34%, 66%, and 45% of CM, CW, and TW, respectively (Table 2). These findings may have implications for PCE risk scores calculated in this analysis given the racial differences by gender. Attempts to incorporate HIV-related metrics (e.g. viral load, CD4) have not yielded any additional fit beyond that provided by the current PCE [24]. A better calibrated tool has yet to be validated in people living with HIV, so the PCE and FRS remain the best available tool for exploring CVD risk.

CNICS is one of the largest clinical cohorts of people living with HIV and to our knowledge, this analysis including 221 TW is the largest cohort study to assess CVD risk and GAHT use among TW living with HIV to date. Yet, since the beginning of follow-up in this cohort (as far back as 1995 for some CNICS sites) there have only been 3 adjudicated myocardial infarctions documented among TW participants. This low incidence of myocardial infarctions could be explained by the younger age of TW compared with CW and CM in the CNICS cohort. Given the limited number of CVD events, this analysis considering multiple predictors of CVD (systolic blood pressure, diabetes, hypertension, cholesterol and smoking) together using the PCE and FRS represents an important first step toward describing the cardiovascular health of TW living with HIV.

Medications captured by CNICS are prescriptions, and may not reflect true medication use. Furthermore, for patients who received care elsewhere prior to enrolling in HIV care in a CNICS clinic, and for patients who receive primary medical care from someone other than their HIV provider, some medications may be missing from the medical record. This may mean that we underestimated years of ART exposure or prior exposure to an abacavir-containing ART regimen. However, history of ART exposure is such an important piece of information when providers decide on future ART regimens that we expect measurement error in these two variables to be minimal. Medication use that is not prescribed, e.g., sex hormones obtained outside of the formal health sector, is not likely to be recorded. Our sample of TW was not powered to stratify by GAHT regimen, dose, or years of exposure, preventing determination of any differential CVD risk by these factors. Further research accounting for these prescription details are a crucial next step in chatacterizing the effects of GAHT on CVD risk.

The ability to identify TW within CNICS allows for more in depth clinical insight than prior studies of CVD among TW living with HIV. Our use of both CW and CM with HIV as control groups allows us to account for variability in sex as well as traditional CVD risk factors. This is even more useful given findings that suggest CM with HIV have higher risk of CVD compared to CW with HIV [46]. The use of multiple imputation with chained equations in this analysis allows for calculation of PCE risk scores and FRS for all participants. This is particularly helpful given the lower number (N = 221) of TW within the study.

Our analysis presents unique findings that establish a baseline for exploring CVD risk among TW living with HIV. CVD among people living with HIV and particularly among TW living with HIV is a growing concern. Key contributors to CVD risk unique to TW living with HIV–such as GAHT, stress and stigma, viral inflammation, and cumulative ART exposure–are not captured by the PCE and FRS. Future investigations of CVD risk among TW living with HIV should collect data on intermediate CVD outcomes (e.g. coronary artery calcium, carotid artery intima-media thickness) or CVD endpoints (e.g. myocardial infarction, stroke) so that actual (versus predicted) CVD morbidity in TW can be quantified.

## Supporting information

S1 Table Medications specific to diabetes considered in CNICS. (DOCX)
