# Supplementary material for: Cardiovascular disease risk among transgender women living with HIV in the United States
Source: PLoS One. 2020 Jul 20;15(7):e0236177. doi: 10.1371/journal.pone.0236177 (PMC7371206; doi:10.1371/journal.pone.0236177)
Supplement: S1 Table — (DOCX) [file pone.0236177.s001.docx]

**Supporting Information 1 – Diabetes Medications in CNICS**

| **Medications specific to diabetes considered in CNICS** |
| --- |
| Metformin + Saxagliptin; Metformin + Sitagliptin; Metformin + Repaglinide; Alogliptin; Linagliptin; Saxagliptin; Sitagliptin; Albiglutide; Dulaglutide; Insulin; Nateglinide; Repaglinide; Pramlintide; Exenatide; Liraglutide; Canagliflozin; Dapagliflozin; Empagliflozin; Chlorpropamide; Glimepiride; Glipizide; Glipizide extended-release; Glyburide; Tolazamide; Tolbutamide; Glyburide + Metformin; Pioglitazone + Glimepiride. Diabetes management medications captured in CNICS are: Acarbose; Miglitol; Metformin; Metformin + Pioglitazone; Metformin + Rosiglitazone; Pioglitazone; Rosiglitazone; Troglitazon |
